# Re-Organization of the Medical Department of the U. S. Army

**Published:** 1862-01

**Authors:** 


### RE-ORGANIZATION OF THE MEDICAL DEPARTMENT OF THE U. S. ARMY.

Bills have been introduced into both Houses of Congress from which it would appear that the following improvements in the administration of the Medical Department of the Army is designed to be instituted. The Senate bill provides for the appointment of the following officers:

"Director-General, with rank of Brigadier-General, who shall be chief of the medical corps, and perform the present Surgeon-General's duties.

Sanitary Inspector-General, with rank of Colonel of Cavalry, who, under the Director-General. shall have general supervision of all that pertains to the sanitary condition of the army.

Six Sanitary Inspectors, with the rank of Lieutenant-Colonel of cavalry, who shall inspect the sanitary condition of the troops, and report to the Sanitary Inspector-General.

Surgeons of first class, with rank of major of cavalry, for staff, hospital and bureau duties.

Fifty surgeons, second class, with rank of captain of cavalry, to be assigned to duty with regiments.

Assistant surgeons, not exceeding seventy, with rank of first lieutenant of cavalry, with duties of assistant surgeons.

Not exceeding seventy-five medical cadets, not less than eighteen nor more than twenty-three years old at their entry, to be examined by a medical board. After three years' continuous service they may be examined for promotion to the rank of the highest class of non-commissioned officers.

As many Hospital Stewards as the service requires, designated by a Sanitary Inspector, on the recommendation of the Senior Surgeon of the post, division, or regiment, with rank of First Sergeants of Cavalry.

Sections 2, 3, 4, 5 and 6, provide for selection by the President, from the whole Army Medical Corps, of suitable persons to fill the places of Director-General, Sanitary Inspector-General, and Sanitary Inspectors, none of whom are to be over sixty years of age. Other officers are to be appointed and promoted by seniority. Vacancies are to be filled from civil life or from Brigade Surgeons of volunteers, after due examination, who are not to be over thirty-five years of age.

Section 5 repeals the allowance of extra rations to Surgeons, upon the completion of ten years' service.

Section 7 provides for the retirement of every medical officer sixty-five years old.

Section 8 repeals all inconsistent laws."

The organization of the Medical Department of the Army has long given just grounds of complaint, and every effort for redress has been indifferently laid aside by the Military Committees in Congress. While we are in the midst of active warfare, with over ten hundred medical officers actively employed by the Government, and nearly an equal number by the revolted territory, it may be the only time when politicians will consider fairly and fully the just claims of the profession, or estimate in any true degree, the value and necessity, of the Medical Department to the safety, strength, and success of the army.

It may be regarded as an unsafe time to make important changes in an organization, while so actively employed as at present, but it seems to us there is little risk in being *just*, while every interest of the Army will be promoted by rightfully acknowledging the dignity, and just claims of the profession. The details of any bill which shall be passed by Congress are of exceeding interest. If appointments cannot be withdrawn from political influences, and made solely with reference to fitness, at least in the medical department, then we have very little ground for expectation that any permanent good can be effected either for the Profession or Army.

To advance men in position on account of the number of years' service, or solely on account of age, is very manifestly inconsistent with efficiency in the service; to do this from political influences, as everything is done in our country, can add neither dignity to the Profession, safety, strength or efficiency to the Army.

